# Standardized Methods for Enhanced Quality and Comparability of Tuberculous Meningitis Studies

**DOI:** 10.1093/cid/ciw757

**Published:** 2016-11-15

**Authors:** Ben J. Marais, Anna D. Heemskerk, Suzaan S. Marais, Reinout van Crevel, Ursula Rohlwink, Maxine Caws, Graeme Meintjes, Usha K. Misra, Nguyen T. H. Mai, Rovina Ruslami, James A. Seddon, Regan Solomons, Ronald van Toorn, Anthony Figaji, Helen McIlleron, Robert Aarnoutse, Johan F. Schoeman, Robert J. Wilkinson, Guy E. Thwaites, for the Tuberculous Meningitis International Research Consortium

**Affiliations:** 1 Marie Bashir Institute for Infectious Diseases and Biosecurity and the Children’s Hospital at Westmead, University of Sydney, Australia; 2 Oxford University Clinical Research Unit, Ho Chi Minh City, Vietnam; 3 Nuffield Department of Medicine, University of Oxford, United Kingdom; 4 Clinical Infectious Diseases Research Initiative, Institute of Infectious Disease and Molecular Medicine and Department of Medicine, University of Cape Town; 5 Department of Neurology, Inkosi Albert Luthuli Central Hospital, Durban, South Africa; 6 Department of Medicine and Radboud Center for Infectious Diseases, Radboud University Medical Center, Nijmegen, The Netherlands; 7 Division of Neurosurgery, University of Cape Town, South Africa; 8 Liverpool School of Tropical Medicine, Liverpool, United Kingdom; 9 Sanjay Ghandi Postgraduate Institute of Medical Sciences, Lucknow, India; 10 Universitas Padjadjaran, Bandung, Indonesia; 11 Department of Medicine, Imperial College London, United Kingdom; 12 Tygerberg Children’s Hospital and the Department of Paediatrics and Child Health, University of Stellenbosch, Tygerberg, South Africa; 13 Francis Crick Institute Mill Hill Laboratory, London, United Kingdom

**Keywords:** tuberculous meningitis, research methods, clinical research, core dataset

## Abstract

Tuberculous meningitis (TBM) remains a major cause of death and disability in tuberculosis-endemic areas, especially in young children and immunocompromised adults. Research aimed at improving outcomes is hampered by poor standardization, which limits study comparison and the generalizability of results. We propose standardized methods for the conduct of TBM clinical research that were drafted at an international tuberculous meningitis research meeting organized by the Oxford University Clinical Research Unit in Vietnam. We propose a core dataset including demographic and clinical information to be collected at study enrollment, important aspects related to patient management and monitoring, and standardized reporting of patient outcomes. The criteria proposed for the conduct of observational and intervention TBM studies should improve the quality of future research outputs, can facilitate multicenter studies and meta-analyses of pooled data, and could provide the foundation for a global TBM data repository.

## Summary

This viewpoint defines a tuberculous meningitis core dataset, including demographic and clinical information, key patient management and monitoring data, and standardized reporting of patient outcomes. Wide adoption of standardized methods will provide a robust evidence base to improve patient outcomes.

Tuberculous meningitis (TBM) was almost universally fatal until the first antibiotic treatment with streptomycin and isoniazid became available [1]. TBM remains a major cause of disease, disability, and death in tuberculosis-endemic areas, but research aimed at improving outcomes is hampered by difficulties in patient recruitment and heterogeneous research methodology. The development of a consensus TBM case definition for use in TBM research has assisted new diagnostic studies by providing a uniform reference standard [2]. However, variable data collection methods, different disease classification systems, and the absence of standardized outcome assessment continue to limit study comparison and complicate efforts to perform systematic reviews and meta-analyses.

The need to standardize clinical trial endpoints is well recognized for pulmonary and drug-resistant tuberculosis research and is the focus of several international consortia (eg, PreDICT-TB [www.predict-tb.eu], RESIST-TB [www.resisttb.org], and TREAT-TB [www.treattb.org]). Core research methods have been proposed for adults and children with multidrug-resistant tuberculosis [3, 4]. In this context, the Oxford University Clinical Research Unit in Vietnam, together with the University of Cape Town’s Clinical Infectious Diseases Research Initiative, organized a meeting of international TBM researchers in Dalat, Vietnam (20–22 May 2015) to assess recent progress and address key challenges in TBM research. Researchers actively engaged in TBM research worldwide were invited with the aim of creating an international consortium that could make recommendations concerning the objectives and methodology of future TBM clinical research.

A TBM research methodological framework was discussed and agreed upon during the meeting by all delegates, broadly defining the key baseline, treatment, and outcome data required in the conduct of TBM research. Thereafter, proposed essential and desirable data were circulated by the lead authors (B. J. M., A. D. H., and G. E. T.) and agreed or adapted by the writing committee (all listed authors) until consensus was found. A statement thereby arose from the meeting, proposing standardized criteria for the conduct and reporting of TBM research and shared data collection templates. We provide an overview of the consensus reached by the consortium, identifying demographic and clinical information to be collected at study enrollment, important aspects related to patient management and monitoring, and standardized reporting of patient outcomes. Specific data points were categorized as either essential or desirable. The essential data points are intended to define minimum criteria for the conduct of both observational and intervention studies, and to identify a core dataset for universal use in future clinical research. Better-harmonized research methods would improve the quality of research outputs and facilitate study comparisons and, in the future, may provide the foundation for a global TBM data repository.

## COHORT DESCRIPTION AND METHODS

Adequate cohort description with clarification of the clinical “point of entry” is essential to ensure study reproducibility and to interrogate differences in study outcomes that may be unrelated to the intervention studied. Because treatment outcomes and diagnostic test performance may vary according to the severity of disease, age, immune status, and patient management, the study population must be well characterized in terms of setting, inclusion criteria, demographics, human immunodeficiency virus (HIV) infection and immune status, disease classification, and treatment received.

## INFORMATION TO COLLECT AT ENROLLMENT

Table 1 provides a summary of essential and desirable baseline information to be collected at study enrollment. Essential data points include information required by the previously published uniform TBM research case definition (Table 2) [2], which should be applied to ensure adequate diagnostic workup and to characterize the study cohort in a standardized fashion. For diagnostic studies, it is important to ensure that control subjects represent a credible clinical entry point for TBM diagnostic evaluation, to assess “real-life” diagnostic accuracy.

**Table 1.** Baseline Information to Be Collected at Enrollment in Tuberculous Meningitis Studies

Information	Essential	Desirable
Demographics	Age (date of birth) Sex	Nationality, ethnicity Medical facility
Presenting symptoms	Neurological symptoms (headache, vomiting, convulsions)—duration^a^ Systemic symptoms (weight loss, night sweats, cough, fever)—duration^a^	
Medical history	Previous and/or current TB Previous TB preventive therapy HIV infection/ART Diabetes (use of insulin) In children BCG vaccination/scar Recent TB contact^a^,^b^	Number of previous TB episodes; date most recent treatment/ preventive therapy; regimen used; adherence History of intravenous drug use BCG vaccination/scar (adults)
Clinical findings	Weight (true or estimated) Glasgow Coma Scale score^a^ Cranial nerve palsy or other focal neurological deficit (specify)^a^ In children Modified Glasgow Coma Scale for infants Head circumference (<5 y) Weight and failure to thrive	Height Neck stiffness Convulsions (focal or generalized) Papilledema or other signs of raised intracranial pressure
Laboratory investigations	CSF Appearance^a^ Total and differential WBC count^a^ Protein and glucose^a^ India ink stain (and/or cryptococcal antigen)^a^ Mycobacterial culture (and/or NAAT) and drug susceptibility testing^a^,^d^ Extraneural samples ZN stain^a^; Mycobacterial culture (and/or NAAT)^a^,^d^ Peripheral blood FBC with differential WBC count Plasma glucose (paired with CSF), sodium, potassium, urea, and creatinine Liver aminotransferases (AST, ALT baseline) HIV test (if not known to be positive) In children TST and/or IGRA^a^	CSF Collection site (lumbar, ventricular) Volume (for TB investigations) ZN stain^c^ Lactate Opening pressure Additional tests to exclude alternative diagnoses (eg, bacterial and fungal culture, enterovirus PCR) IGRA or TST (adults) Hepatitis B or C coinfection Syphilis serology
Imaging	Chest radiograph Signs of active TB^a^; miliary appearance Brain CT or MRI^a^ Hydrocephalus; basal meningeal enhancement, infarct, tuberculoma	Imaging (MRI/CT/ultrasound) of extraneural sites suspected of TB disease^a^ Air encephalogram to differentiate communicating and noncommunicating hydrocephalus Hydrocephalus description; presence of periventricular edema; herniation Infarct; type; single/multiple; anatomical location
Diagnostic certainty and disease severity	Definite, probable, possible or not TBM^a^ BMRC TBM severity grade^e^ (1, 2, or 3)	
If HIV infected	WHO clinical disease staging CD4 count ART	CD4 count (most recent and nadir) HIV RNA load (most recent and highest) Detail of ART regimen

Abbreviations: ALT, alanine aminotransferase; ART, antiretroviral therapy; AST, aspartate aminotransferase; BMRC, British Medical Research Council; CSF, cerebrospinal fluid; CT, computed tomography; FBC, full blood count; HIV, human immunodeficiency virus; IGRA, interferon-γ release assay; MRI, magnetic resonance imaging; NAAT, nucleic acid amplification test (including GeneXpert MTB/RIF); PCR, polymerase chain reaction; TB, tuberculosis; TBM, tuberculous meningitis; TST, tuberculin skin test; WBC, white blood cell; WHO, World Health Organization; ZN, Ziehl-Neelsen.

a Data required for uniform TBM research case definition criteria (Table 2).

b Close/household contact with an infectious (pulmonary) TB case during the past year.

c The yield of CSF ZN microscopy is so low that many laboratories do not offer this as a routine test.

d Genotypic (at least GeneXpert MTB/RIF) or phenotypic drug susceptibility testing must be performed if *Mycobacterium tuberculosis* is detected.

e According to modified BMRC criteria.

**Table 2.** Uniform Tuberculous Meningitis Research Case Definition Criteria[^2^]

Criteria	
Clinical criteria (maximum category score = 6)	
Symptom duration of >5 d	4
Systemic symptoms suggestive of TB (≥1): weight loss/(poor weight gain in children), night sweats, or persistent cough >2 wk	2
History of recent close contact with an individual with pulmonary TB or a positive TST/IGRA in a child aged <10 y	2
Focal neurological deficit (excluding cranial nerve palsies)	1
Cranial nerve palsy	1
Altered consciousness	1
CSF criteria (maximum category score = 4)	
Clear appearance	1
Cells: 10–500/µL	1
Lymphocytic predominance (>50%)	1
Protein concentration >1 g/L	1
CSF to plasma glucose ratio of <50% or an absolute CSF glucose concentration <2.2 mmol/L	1
Cerebral imaging criteria (maximum category score = 6)	
Hydrocephalus (CT and/or MRI)	1
Basal meningeal enhancement (CT and/or MRI)	2
Tuberculoma (CT and/or MRI)	2
Infarct (CT and/or MRI)	1
Precontrast basal hyperdensity (CT)	2
Evidence of tuberculosis elsewhere (maximum category score = 4)	
Chest radiograph suggestive of active TB (excludes miliary TB)	2
Chest radiograph suggestive of miliary TB	4
CT/MRI/US evidence for TB outside the CNS	2
AFB identified or *Mycobacterium tuberculosis* cultured from another source (ie, sputum, lymph node, gastric washing, urine, blood culture)	4
Positive commercial *M. tuberculosis* NAAT from extraneural specimen	4
Exclusion of alternative diagnoses: An alternative diagnosis must be confirmed microbiologically, serologically, or histopathologically.	
Definite TBM: AFB seen on CSF microscopy, positive CSF *M. tuberculosis* culture, or positive CSF *M. tuberculosis* commercial NAAT in the setting of symptoms/signs suggestive of meningitis; or AFB seen in the context of histological changes consistent with TB brain or spinal cord together with suggestive symptoms/signs and CSF changes, or visible meningitis (on autopsy).	
Probable TBM: total score of ≥12 when neuroimaging available or total score of ≥10 when neuroimaging unavailable. At least 2 points should either come from CSF or cerebral imaging criteria.	
Possible TBM: total score of 6–11 when neuroimaging available, or total score of 6–9 when neuroimaging unavailable.	

Abbreviations: AFB, acid-fast bacilli; CNS, central nervous system; CSF, cerebrospinal fluid; CT, computed tomography; IGRA, interferon-γ release assay; MRI, magnetic resonance imaging; NAAT, nucleic acid amplification test; TB, tuberculosis; TBM, tuberculous meningitis; TST, tuberculin skin test; US, ultrasound.

### Disease Severity and Phenotype

Given the diversity of clinical presentation and disease severity, it is important to grade TBM severity in a pragmatic and standardized fashion. As a minimum, HIV status (preferably with CD4 count and World Health Organization [WHO] clinical disease staging) must be recorded and the modified British Medical Research Council (BMRC) TBM grade should be ascertained in all studied patients before the start of treatment. BMRC investigators [1] first graded TBM patients as “early” (no clinical signs of meningitis or focal neurology and fully conscious); “medium” (patient’s condition falling between early and advanced); and “advanced” (extremely ill, in deep coma). With the introduction of the Glasgow Coma Scale (GCS) in 1974 [5], this was modified as grade I (GCS 15; no focal neurological signs), grade II (GCS 11–14, or 15 with focal neurological signs), and grade III (GCS ≤10) disease [6]. Numerous studies across all age groups have shown that the modified BMRC grade is a strong independent predictor of outcome [6–10]. TBM patients with grade I disease are often underrepresented in studies, as their nonspecific symptoms may not trigger a lumbar puncture, which usually provides the entry point for TBM studies. Subdivision of grade II disease has been proposed [11], as have other prognostic systems based on weighted scoring of mental status, seizures, cranial nerve palsies, motor deficit, and tone [12], but these have not been validated. Because level of consciousness is influenced by rapidly reversible raised intracranial pressure and electrolyte disturbances, it is important to repeat the BMRC disease severity grading 7 days after TBM treatment initiation. This provides a useful reassessment of disease severity that may be better associated with long-term outcome than a single baseline assessment.

### Baseline Investigations

Essential and desired study investigations, to be performed at enrollment, are summarized in Table 1. Cerebrospinal fluid (CSF) sampling and analysis are essential to assist diagnostic workup and cohort description and to define prognosis. Low CSF white blood cell count, low glucose, and high lactate have been associated with death in studies from Vietnam [13]. In HIV-coinfected patients, baseline CSF neutrophil count and culture positivity for *Mycobacterium tuberculosis* are predictive of TBM immune reconstitution inflammatory syndrome (IRIS) [14]. Cryptococcal meningitis and TBM have similar presenting features and CSF cryptococcal antigen tests should be performed, especially in those with advanced HIV infection (peripheral CD4^+^ count <100 cells/µL), in whom both diseases are common. Peripheral blood findings have limited diagnostic or prognostic value, but it is important to document anemia, determine baseline renal and liver function tests for drug toxicity monitoring, and assess HIV and immune status. Hyponatremia has been linked to a worse outcome [15], while in HIV-1–coinfected patients lower blood hematocrit [13] and low CD4^+^ T-cell count [16, 17] have been associated with death.

Brain computed tomography (CT) with or without contrast and magnetic resonance imaging (MRI) characterize the pathological processes underlying the clinical presentation, disease course, and long-term consequences of TBM. Baseline brain imaging is recommended for all patients, although this may not be available in all settings [18]. Classic imaging findings include basal meningeal enhancement, hydrocephalus, tuberculomas, and cerebral infarction [19]. MRI is more sensitive in detecting early ischemia and brainstem lesions [20]. TBM-related infarcts are most commonly located in the territories of the proximal middle cerebral artery and the medial lenticulostriate and thalamoperforating vessels [21, 22]. Brain imaging provides a window on the pathophysiology of TBM, and standardized documentation of these complications and their response to treatment could improve management and may suggest new therapeutic approaches.

### Sample Collection and Laboratory Methods

Diagnostic yield is influenced by both the type and quality of specimens collected; therefore careful description of specimen collection methods and test procedures are important. Microbiological yield is affected by CSF volume, sample transport delays, and processing techniques [23]. The CSF sample volume used for each mycobacterial diagnostic test should be reported. In addition, adequate quality assurance of all research laboratories is essential to ensure test reliability.

## MANAGEMENT AND MONITORING

**Figure 1.**
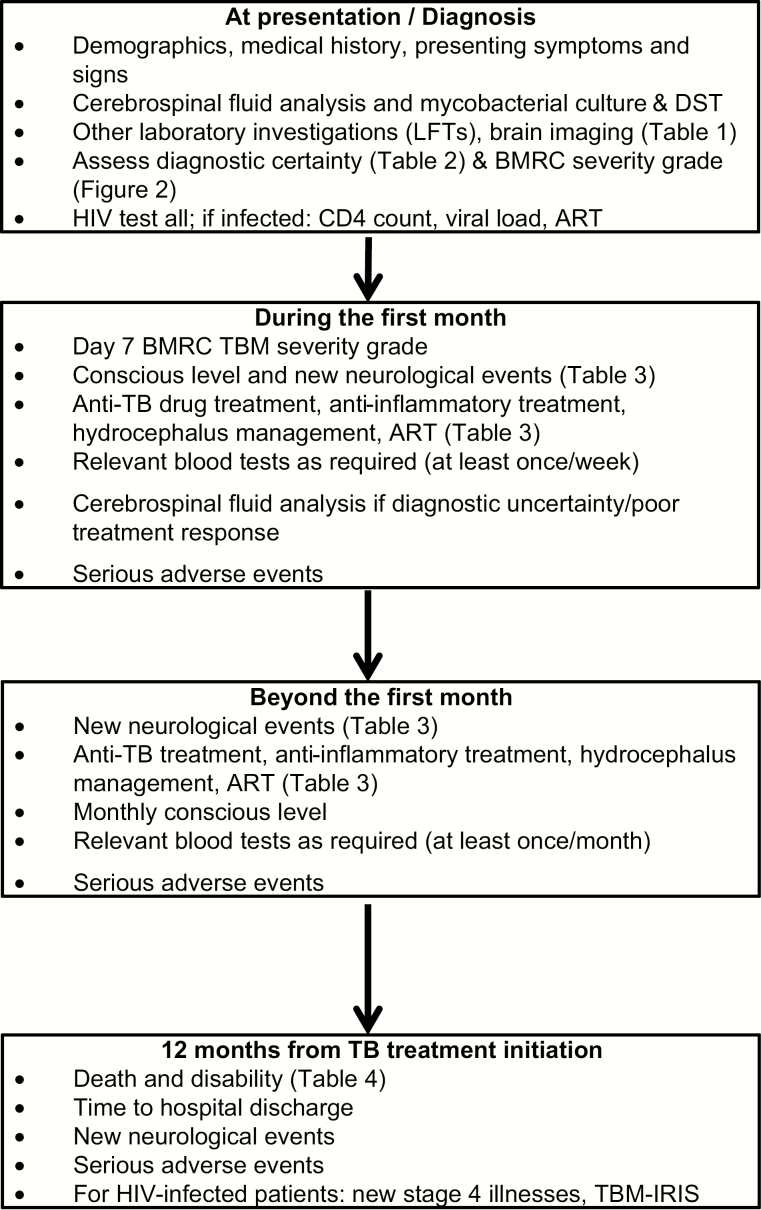
Proposed minimum schedule of investigations and outcome measurements in studies of tuberculous meningitis. Abbreviations: ART, antiretroviral therapy; BMRC, British Medical Research Council; DST, drug susceptibility testing; HIV, human immunodeficiency virus; IRIS, immune reconstitution inflammatory syndrome; LFTs, liver function tests; TBM, tuberculous meningitis.

Ensuring minimal standards of ancillary care and patient monitoring is essential for ethical reasons and for reporting purposes, as differences in local management practices are important confounders when assessing outcomes. Management and monitoring protocols should be adequately described, including (1) antituberculosis drug treatment; (2) adjunctive anti-inflammatory therapy; (3) management of hydrocephalus; and (4) other supportive care (Table 3, Figure 1).

**Table 3.** Data Collection Requirements for Patient Management and Monitoring in Tuberculous Meningitis Studies

Requirements	Essential	Desirable
Management	TB treatment Initiation date Drug doses and route of administration Treatment duration and adherence Anti-inflammatory treatment Corticosteroids and/or other anti-inflammatory agents (type, dose, duration) Hydrocephalus management Medical (drugs, dose, duration) Surgical (shunt type) ART Initiation date (if new) Regimen used Treatment adherence Time from TBM treatment to ART initiation	TB treatment–related adverse events (especially drug-induced liver injury) Surgery-related adverse events Description of adverse events Time to shunt ART-related adverse effects Treatment interruptions (number, total duration)
Monitoring	Observations^a^ Level of consciousness Change in TBM severity grade (day 7) Blood tests^c^ Full blood count Serum sodium, potassium, urea, and creatinine Liver aminotransferases (ALT, AST)	CSF ^b^ Opening pressure; cell count and differential; protein, glucose; Ziehl-Neelsen stain; and mycobacterial culture and DST During acute illness^a^ record lowest blood pressure, pulse oximetry, blood glucose If admitted to ICU Blood gas; continuous intracranial pressure and cerebral oxygenation/ perfusion monitoring If HIV infected CD4 T-cell count at 3, 6, and 12 months HIV RNA load at 6 and 12 months

Abbreviations: ALT, alanine aminotransferase; ART, antiretroviral therapy; AST, aspartate aminotransferase; CSF, cerebrospinal fluid; DST, drug susceptibility testing; HIV, human immunodeficiency virus; ICU, intensive care unit; TB, tuberculosis; TBM, tuberculous meningitis.

a At a minimum, clinical observations should be performed daily during the first 7 days, weekly during the first month, and monthly during the first 6 months.

b If the diagnosis of TBM is uncertain, it is recommended to repeat CSF analysis 3–7 days after the start of treatment. Otherwise, CSF after 30 and 60 days of treatment can help to assess treatment response and likelihood of drug resistance.

c As a minimum, blood tests should be performed at diagnosis, weekly during the first month, and monthly during the first 6 months.

### Antituberculosis Drug Treatment

As summarized in Table 3, it is essential to document the dose, route of administration, and duration of all antituberculosis drugs used in TBM treatment. Antituberculosis drug–related adverse events are more important in the treatment of TBM than other forms of tuberculosis because treatment interruptions have been independently associated with death [24]. Drug-induced liver injury (DILI) is the commonest adverse event and should be documented alongside changes in antituberculosis drug regimen. Outstanding questions remain concerning optimal management when DILI occurs, and harmonized data collection would allow analyses to address these.

When performing antituberculosis drug treatment trials, it is important to document drug quality as this can be highly variable. WHO-prequalified drugs have been rigorously evaluated and meet strict quality criteria (a list of prequalified drugs is available at http://apps.who.int/prequal/query/ProductRegistry.aspx). For other drugs, pharmaceutical companies should provide “certificates of analysis” and data on bioequivalence.

### Pharmacokinetic/Pharmacodynamic Substudies

Pharmacokinetic/pharmacodynamic (PK/PD) substudies can help to explain trial findings and provide important dosing information for future studies. Evaluation of individual exposures achieved provides insight into predictors of drug exposure and enables concentration-response relationships (PK/PD analysis) to be established [25–27]; the latter may reveal exposure thresholds predictive of good treatment outcome or drug toxicity [26, 28, 29]. Pharmacokinetic analysis should ideally include both plasma and CSF measurements, although CSF concentrations may not reflect the brain tissue concentration of highly lipophilic drugs [30, 31]. Pharmacokinetic studies often take place at “steady state,” when the processes of accumulation or induction are complete (in rifampicin this can take up to 10 days), but in TBM we suggest measuring exposures during the critical first days of treatment when mortality is highest [27]. CSF sampling after weeks of treatment may yield different results, as CSF drug penetration may reduce as meningeal inflammation lessens.

The standard method to assess CSF penetration is the CSF to plasma ratio for total drug exposure (area under the concentration-time curve [AUC]) during the dosing interval, which requires multiple plasma and CSF samples [25]. Alternatively, a CSF to plasma concentration ratio can be established by a single point measurement, but this is time dependent as the ratio is often variable over the dosing interval [31]. Although single time point CSF to plasma ratios should be interpreted with caution, pharmacokinetic modeling may approximate the CSF to plasma AUC ratio despite limited sampling, if such sampling takes place at multiple time points. It is important that CSF to plasma concentration ratios should be based upon estimated protein-unbound (“free”) exposure measures. In plasma, only the protein-unbound fraction is active and able to penetrate into the CSF. If a drug with high protein binding in plasma has excellent CSF penetration, a CSF to plasma ratio based on protein-unbound concentrations would be close to unity. In contrast, a ratio based on total (bound + unbound) concentrations would incorrectly suggest poor penetration [31], as is the case with rifampicin. Therefore, a correction for protein binding of plasma concentrations should always be made [25, 26].

Pharmacokinetic sampling can be “intensive” or “sparse,” or ideally a mixture of both to assist accurate modeling. Analytical methods used to determine drug concentrations should have appropriate intralaboratory (internal) assessment of accuracy, precision, and other validation measures. Participation in an interlaboratory (external) proficiency testing program is recommended [32]. Of note, CSF drug concentrations cannot be measured using plasma assays without careful validation.

### Adjunctive Anti-inflammatory Therapy

TBM studies should describe the type, dose, route of administration, and duration of anti-inflammatory therapy used (Table 3). Adjunctive corticosteroids are currently recommended for all HIV-uninfected TBM patients during the first 6–8 weeks of treatment [9]; they are also used in HIV-infected patients, although the evidence of benefit is much less clear. Corticosteroids are also often used for the management of tuberculomas and IRIS in HIV-infected patients, although the evidence base is weak. Some patients who do not respond to corticosteroids may benefit from other agents, such as thalidomide or anti–tumor necrosis factor-α biologic agents [33–36]. Two small studies also suggest that aspirin may reduce cerebral infarcts [37, 38], but this has not been confirmed in larger-scale studies.

### Management of Hydrocephalus

As a minimum, the presence of hydrocephalus assessed by brain CT or MRI should be documented at the start of treatment. An assessment of whether the hydrocephalus is communicating or noncommunicating is desirable, and the management (medical or surgical) should be documented (Table 3). Raised intracranial pressure, largely due to hydrocephalus, is a common problem in patients with TBM [10, 39]. If untreated, hydrocephalus can exacerbate the cerebral ischemia caused by perfusion-limiting vasculitis, which is a key feature of TBM. Untreated hydrocephalus is independently associated with death [40, 41]. Whether the hydrocephalus is communicating or noncommunicating [10, 42] has important management implications. At present, the only way to reliably differentiate communicating from noncommunicating hydrocephalus is with an air encephalogram or using contrast ventriculography [43, 44]. Performing an air encephalogram does not require any special resources and can be performed when collecting a CSF sample. Ventricular shunting is usually indicated for noncommunicating hydrocephalus, while a combination of diuretics (acetazolamide and furosemide) may treat communicating hydrocephalus [45]; the value of this approach requires further confirmation in adults.

### General Supportive Care

The provision of optimal supportive care in patients with TBM is rarely reported and often neglected and is therefore highlighted here. Hyponatremia occurs in a high percentage of TBM patients, is associated with poorer outcome [10], and should be documented (Table 3). Hyponatremia may result from inappropriate antidiuretic hormone secretion or cerebral salt wasting or may represent an appropriate compensatory response to maintain cerebral perfusion. Plasma sodium concentrations should be recorded at baseline and through the early phase of hospital treatment. In critically ill patients, changes in cerebral perfusion and oxygenation are highly dynamic, but can be captured through continuous intracranial monitoring. This is invasive and only possible during intensive care admission in settings where such facilities exist. Transcranial Doppler [46] and near-infrared spectroscopy provide less-invasive alternatives, but these methods require more rigorous validation and have not yet demonstrated clinical utility. Simple measures to reduce cerebral ischemia and brain cell metabolic stress include maintaining adequate blood pressure and glucose levels, providing supplemental oxygen, and controlling fever [47]. These parameters should be recorded in all critically ill patients (Table 3).

## OUTCOME MEASURES

The inconsistent reporting of outcome measures limits critical study evaluation and comparison. TBM is associated with high mortality; therefore, death is an essential outcome measure. The time of death in relation to the start of antituberculosis treatment should also be documented, and we recommend reporting to at least 12 months from antituberculosis treatment initiation. Cause of death is notoriously difficult to determine without formal postmortem examination, but deaths directly attributable to TBM are more likely to occur in the first 3 months of treatment; later deaths may be caused by secondary infections, for example, especially in those left with severe neurological disability. An assessment by the attending physician as to the likely cause of death (TBM attributable/not attributable) is desirable but not essential, given the inherent limitations of this approach.

The reporting of functional outcomes are also essential, but different measures are used [9], and detailed neurocognitive outcomes are rarely assessed [48]. Given the importance of neurodisability and comparable outcome measurement, we recommend that the Modified Rankin Score should be recorded 12 months from antituberculosis treatment initiation in all adults and in children. The score (detailed in the Supplementary Appendix) assesses whether or not the subject can live independently of others. We also recommend recording the Pediatric Version of the Glasgow Outcome Scale–Extended (GOS-E peds) in children (Table 4); however, this scale, created for children following neurotrauma, needs further validation in childhood TBM.

**Table 4.** Primary and Secondary Outcome Measures to Be Reported in Tuberculous Meningitis Studies

All Patients	Essential	Desirable
Primary outcomes (assessed 12 mo from start of anti-TB treatment)	Death; time to death Neurological disability^a^	Cause of death^b^ Detailed neurocognitive and behavioral outcomes
Secondary outcomes	Coma clearance^c^; time to event New neurological event^d^; time to event Change in TBM severity grade by day 7 of anti-TB treatment	Coma management Radiological findings (brain imaging) investigating new neurological event
	All serious adverse events^e^ and their relationship to the disease or drugs given	Anti-TB drug treatment interruptions (number, total duration) Time to event; likely causative agent; management Complications related to corticosteroid therapy
	Time to hospital discharge	Admission to ICU; duration of admission Requires mechanical ventilation; duration of ventilation
HIV-infected patients		
New stage 4 illnesses	Nature of condition; time to event	Management of condition
Neurological TB-IRIS	Nature of condition; time to event Report TB-IRIS criteria used	Management of condition

Abbreviations: HIV, human immunodeficiency virus; ICU, intensive care unit; IRIS, immune reconstitution inflammatory syndrome; TB, tuberculosis; TBM, tuberculous meningitis.

a Use Modified Rankin Score in adults and children, and the GOS-E (pediatric version of the Glasgow Outcome Scale–Extended) score in children (see Supplementary Data for scoring criteria).

b Cause of death should be reported as TBM attributable or not attributable (determined by postmortem and/or clinical records).

c From TBM treatment initiation until Glasgow Coma Scale (GCS) score of 15 for 2 consecutive days.

d Defined as a fall in GCS of ≥2 points for ≥48 hours, new focal neurological sign, or new onset of seizures.

e Any adverse event, adverse reaction, or unexpected adverse reaction that results in death, is life threatening, requires hospitalization or prolongation of existing hospitalization, results in persistent or significant disability or incapacity, or consists of a congenital anomaly or birth defect.

TBM causes significant long-term neurocognitive impairment in children [10, 49] and adults [7], and detailed neurocognitive and psychiatric outcomes should be reported where possible. The Griffiths Mental Developmental Scales or the Pediatric Cerebral Performance Category Scale provide assessment on age-appropriate neurocognitive and developmental outcomes. It is important that children are compared to age-matched controls from the same socioeconomic background, given major environmental influences on early cognitive development.

### Paradoxical Reactions

Clinical deterioration after the start of antituberculosis treatment—commonly called paradoxical reactions and associated with increased intracerebral inflammation—occurs in approximately 30% of HIV-uninfected individuals with TBM [50] and around 50% of those who are HIV infected [14]. Paradoxical reactions are associated with new or worsening intracerebral tuberculomas, hydrocephalus, infarcts, and/or spinal radiculomyelitis. In HIV-infected subjects recently started on antiretroviral therapy, these events may be defined as TBM- IRIS following the International Network for the Study of HIV-Associated IRIS criteria [51], modified for TBM.

All suspected paradoxical reactions and their timing with respect to antituberculosis drug initiation should be recorded (Table 4). When possible, providers should investigate all suspected paradoxical reactions with brain imaging and document the findings, management given, and outcomes. Alternative causes that should be excluded as far as possible include drug resistance, poor adherence to treatment, drug-related adverse events, and other opportunistic infections. We also recommend that all WHO HIV stage 4 illnesses should be recorded throughout the course of TBM treatment (Table 4).

## PROPOSED CORE DATASET

Participants at the workshop reinforced calls for standardized approaches, including laboratory and clinical assessment procedures and data reporting. All laboratory tests should be guided by detailed standard operating procedures, including sample collection, processing, transport, storage, and laboratory procedures for specific diagnostic tests, including quality assurance measures; relevant standard operating procedures are included in the Supplementary Data. Demographic and clinical data should also be collected in a standardized fashion. The essential elements listed in the tables provide the basis for a core dataset that represents the minimum data to be captured in future TBM studies. A proposed data capture form that includes all the proposed “essential” and “desirable” variables (Tables 1–4) is included in the Supplementary Data.

## CONCLUSIONS

Developing standardized approaches represents a critical first step to establish the evidence base required to improve TBM detection and outcome. Poor study comparability due to variable methods, case definitions, and data collection and reporting highlight the inadequacy of current approaches. Wide adoption of the standard methods proposed here should help to move the field forward and ensure that the benefits of technological advances are fully realized. This document should be viewed as a living tool that will be refined as the evidence base and field experience with conducting multicenter TBM studies grow and are critically evaluated at future meetings.

## Supplementary Material

Supplementary Data

## Notes

***Acknowledgments.*** The Tuberculous Meningitis International Meeting was convened by the Oxford University Clinical Research Unit in Vietnam and the Clinical Infectious Diseases Research Initiative of the University of Cape Town, with support from the Li Ka Shing Foundation and Wellcome Trust, UK.

***Potential conflicts of interest.*** All authors: No potential conflicts. All authors have submitted the ICMJE Form for Disclosure of Potential Conflicts of Interest. Conflicts that the editors consider relevant to the content of the manuscript have been disclosed.

## References

[1] Medical Research Council. Streptomycin treatment of tuberculous meningitis. Br Med J 1948: 582–97.20787409

[2] Marais S Thwaites G Schoeman JF Tuberculous meningitis: a uniform case definition for use in clinical research. Lancet Infect Dis 2010; 10:803–12. 20822958 10.1016/S1473-3099(10)70138-9

[3] Furin J Alirol E Allen E Drug-resistant tuberculosis clinical trials: proposed core research definitions in adults. Int J Tuberc Lung Dis 2016; 20:290–4. 27046707 10.5588/ijtld.15.0490 PMC4843774

[4] Seddon JA Perez-Velez CM Schaaf HS; Sentinel Project on Pediatric Drug-Resistant Tuberculosis Consensus statement on research definitions for drug-resistant tuberculosis in children. J Pediatric Infect Dis Soc 2013; 2:100–9. 23717785 10.1093/jpids/pit012 PMC3665326

[5] Teasdale G Jennett B Assessment of coma and impaired consciousness. A practical scale. Lancet 1974; 2:81–4. 4136544 10.1016/s0140-6736(74)91639-0

[6] Thwaites GE Tran TH Tuberculous meningitis: many questions, too few answers. Lancet Neurol 2005; 4:160–70. 15721826 10.1016/S1474-4422(05)01013-6

[7] Kalita J Misra UK Ranjan P Predictors of long-term neurological sequelae of tuberculous meningitis: a multivariate analysis. Eur J Neurol 2007; 14:33–7. 17222110 10.1111/j.1468-1331.2006.01534.x

[8] Misra UK Kalita J Roy AK Mandal SK Srivastava M Role of clinical, radiological, and neurophysiological changes in predicting the outcome of tuberculous meningitis: a multivariable analysis. J Neurol Neurosurg Psychiatry 2000; 68:300–3. 10675210 10.1136/jnnp.68.3.300 PMC1736823

[9] Thwaites GE Nguyen DB Nguyen HD Dexamethasone for the treatment of tuberculous meningitis in adolescents and adults. N Engl J Med 2004; 351:1741–51. 15496623 10.1056/NEJMoa040573

[10] van Well GT Paes BF Terwee CB Twenty years of pediatric tuberculous meningitis: a retrospective cohort study in the western cape of South Africa. Pediatrics 2009; 123:e1–8. 19367678 10.1542/peds.2008-1353

[11] Wolzak NK Cooke ML Orth H van Toorn R The changing profile of pediatric meningitis at a referral centre in Cape Town, South Africa. J Trop Pediatr 2012; 58:491–5. 22791086 10.1093/tropej/fms031

[12] Saitoh A Pong A Waecker NJ Jr Leake JA Nespeca MP Bradley JS Prediction of neurologic sequelae in childhood tuberculous meningitis: a review of 20 cases and proposal of a novel scoring system. Pediatr Infect Dis J 2005; 24:207–12. 15750455 10.1097/01.inf.0000154321.61866.2d

[13] Thwaites GE Duc Bang N Huy Dung N The influence of HIV infection on clinical presentation, response to treatment, and outcome in adults with tuberculous meningitis. J Infect Dis 2005; 192:2134–41. 16288379 10.1086/498220

[14] Marais S Meintjes G Pepper DJ Frequency, severity, and prediction of tuberculous meningitis immune reconstitution inflammatory syndrome. Clin Infect Dis 2013; 56:450–60. 23097584 10.1093/cid/cis899 PMC3540040

[15] Torok ME Chau TT Mai PP Clinical and microbiological features of HIV-associated tuberculous meningitis in Vietnamese adults. PLoS One 2008; 3:e1772. 18350135 10.1371/journal.pone.0001772 PMC2262136

[16] Simmons CP Thwaites GE Quyen NT Pretreatment intracerebral and peripheral blood immune responses in Vietnamese adults with tuberculous meningitis: diagnostic value and relationship to disease severity and outcome. J Immunol 2006; 176:2007–14. 16424233 10.4049/jimmunol.176.3.2007

[17] Marais S Pepper DJ Schutz C Wilkinson RJ Meintjes G Presentation and outcome of tuberculous meningitis in a high HIV prevalence setting. PLoS One 2011; 6:e20077. 21625509 10.1371/journal.pone.0020077 PMC3098272

[18] Thwaites G Fisher M Hemingway C Scott G Solomon T Innes J; British Infection Society British Infection Society guidelines for the diagnosis and treatment of tuberculosis of the central nervous system in adults and children. J Infect 2009; 59:167–87. 19643501 10.1016/j.jinf.2009.06.011

[19] Schoeman JF Van Zyl LE Laubscher JA Donald PR Serial CT scanning in childhood tuberculous meningitis: prognostic features in 198 cases. J Child Neurol 1995; 10:320–9. 7594269 10.1177/088307389501000417

[20] Figaji AA Sandler SI Fieggen AG Le Roux PD Peter JC Argent AC Continuous monitoring and intervention for cerebral ischemia in tuberculous meningitis. Pediatr Crit Care Med 2008; 9:e25–30. 18843248 10.1097/PCC.0b013e318172e8b7

[21] Andronikou S Wilmshurst J Hatherill M VanToorn R Distribution of brain infarction in children with tuberculous meningitis and correlation with outcome score at 6 months. Pediatr Radiol 2006; 36:1289–94. 17031634 10.1007/s00247-006-0319-7

[22] Kalita J Misra UK Nair PP Predictors of stroke and its significance in the outcome of tuberculous meningitis. J Stroke Cerebrovasc Dis 2009; 18:251–8. 19560677 10.1016/j.jstrokecerebrovasdis.2008.11.007

[23] Thwaites GE Chau TT Farrar JJ Improving the bacteriological diagnosis of tuberculous meningitis. J Clin Microbiol 2004; 42:378–9. 14715783 10.1128/JCM.42.1.378-379.2004 PMC321694

[24] Thwaites GE Nguyen DB Nguyen HD Dexamethasone for the treatment of tuberculous meningitis in adolescents and adults. N Engl J Med 2004; 351:1741–51. 15496623 10.1056/NEJMoa040573

[25] Alffenaar JW van Altena R Bökkerink HJ Pharmacokinetics of moxifloxacin in cerebrospinal fluid and plasma in patients with tuberculous meningitis. Clin Infect Dis 2009; 49:1080–2. 19712035 10.1086/605576

[26] Thwaites GE Bhavnani SM Chau TT Randomized pharmacokinetic and pharmacodynamic comparison of fluoroquinolones for tuberculous meningitis. Antimicrob Agents Chemother 2011; 55:3244–53. 21502621 10.1128/AAC.00064-11 PMC3122453

[27] Ruslami R Ganiem AR Dian S Intensified regimen containing rifampicin and moxifloxacin for tuberculous meningitis: an open-label, randomised controlled phase 2 trial. Lancet Infect Dis 2013; 13:27–35. 23103177 10.1016/S1473-3099(12)70264-5

[28] Te Brake L Dian S Ganiem AR Pharmacokinetic/pharmacodynamic analysis of an intensified regimen containing rifampicin and moxifloxacin for tuberculous meningitis. Int J Antimicrob Agents 2015; 45:496–503. 25703312 10.1016/j.ijantimicag.2014.12.027

[29] Savic RM Ruslami R Hibma JE Pediatric tuberculous meningitis: model-based approach to determining optimal doses of the anti-tuberculosis drugs rifampin and levofloxacin for children. Clin Pharmacol Ther 2015; 98:622–9. 26260983 10.1002/cpt.202 PMC4888594

[30] Donald PR Cerebrospinal fluid concentrations of antituberculosis agents in adults and children. Tuberculosis (Edinb) 2010; 90:279–92. 20709598 10.1016/j.tube.2010.07.002

[31] Hoetelmans RM Sanctuary sites in HIV-1 infection. Antivir Ther 1998; 3(suppl 4):13–7. 10723504

[32] Aarnoutse RE Sturkenboom MG Robijns K An interlaboratory quality control programme for the measurement of tuberculosis drugs. Eur Respir J 2015; 46:268–71. 25882800 10.1183/09031936.00177014

[33] Schoeman JF Springer P van Rensburg AJ Adjunctive thalidomide therapy for childhood tuberculous meningitis: results of a randomized study. J Child Neurol 2004; 19:250–7. 15163089 10.1177/088307380401900402

[34] Schoeman JF Andronikou S Stefan DC Freeman N van Toorn R Tuberculous meningitis-related optic neuritis: recovery of vision with thalidomide in 4 consecutive cases. J Child Neurol 2010; 25:822–8. 20519667 10.1177/0883073809350507

[35] Coulter JB Baretto RL Mallucci CL Tuberculous meningitis: protracted course and clinical response to interferon-gamma. Lancet Infect Dis 2007; 7:225–32. 17317604 10.1016/S1473-3099(07)70054-3

[36] Lee JY Yim JJ Yoon BW Adjuvant interferon-γ treatment in two cases of refractory tuberculosis of the brain. Clin Neurol Neurosurg 2012; 114:732–4. 22209143 10.1016/j.clineuro.2011.12.013

[37] Misra UK Kalita J Nair PP Role of aspirin in tuberculous meningitis: a randomized open label placebo controlled trial. J Neurol Sci 2010; 293:12–7. 20421121 10.1016/j.jns.2010.03.025

[38] Schoeman JF Janse van Rensburg A Laubscher JA Springer P The role of aspirin in childhood tuberculous meningitis. J Child Neurol 2011; 26:956–62. 21628697 10.1177/0883073811398132

[39] Thwaites GE Macmullen-Price J Tran TH Serial MRI to determine the effect of dexamethasone on the cerebral pathology of tuberculous meningitis: an observational study. Lancet Neurol 2007; 6:230–6. 17303529 10.1016/S1474-4422(07)70034-0 PMC4333204

[40] Clemente Morgado T Kinsky M Carrara H Rothemeyer S Semple P Prognostic value of computed tomography-evident cerebral infarcts in adult patients with tuberculous meningitis and hydrocephalus treated with an external ventricular drain. World Neurosurg 2013; 80:e255–60. 23041069 10.1016/j.wneu.2012.09.021

[41] Hsu PC Yang CC Ye JJ Huang PY Chiang PC Lee MH Prognostic factors of tuberculous meningitis in adults: a 6-year retrospective study at a tertiary hospital in northern Taiwan. J Microbiol Immunol Infect 2010; 43:111–8. 20457427 10.1016/S1684-1182(10)60018-7

[42] Lamprecht D Schoeman J Donald P Hartzenberg H Ventriculoperitoneal shunting in childhood tuberculous meningitis. Br J Neurosurg 2001; 15:119–25. 11360374 10.1080/02688690020036801

[43] Bruwer GE Van der Westhuizen S Lombard CJ Schoeman JF Can CT predict the level of CSF block in tuberculous hydrocephalus? Childs Nerv Syst 2004; 20:183–7. 14968373 10.1007/s00381-003-0887-x

[44] Figaji AA Fieggen AG Peter JC Air encephalography for hydrocephalus in the era of neuroendoscopy. Childs Nerv Syst 2005; 21:559–65. 15714352 10.1007/s00381-004-1119-8

[45] Schoeman J Donald P van Zyl L Keet M Wait J Tuberculous hydrocephalus: comparison of different treatments with regard to ICP, ventricular size and clinical outcome. Dev Med Child Neurol 1991; 33:396–405. 2065826 10.1111/j.1469-8749.1991.tb14899.x

[46] van Toorn R Schaaf HS Solomons R Laubscher JA Schoeman JF The value of transcranial Doppler imaging in children with tuberculous meningitis. Childs Nerv Syst 2014; 30:1711–6. 24828794 10.1007/s00381-014-2435-2

[47] Figaji AA Fieggen AG The neurosurgical and acute care management of tuberculous meningitis: evidence and current practice. Tuberculosis (Edinb) 2010; 90:393–400. 20970381 10.1016/j.tube.2010.09.005

[48] Chen HL Lu CH Chang CD Structural deficits and cognitive impairment in tuberculous meningitis. BMC Infect Dis 2015; 15:279. 26198732 10.1186/s12879-015-1011-z PMC4510907

[49] Schoeman J Wait J Burger M Long-term follow up of childhood tuberculous meningitis. Dev Med Child Neurol 2002; 44:522–6. 12206617 10.1017/s0012162201002493

[50] Singh AK Malhotra HS Garg RK Paradoxical reaction in tuberculous meningitis: presentation, predictors and impact on prognosis. BMC Infect Dis 2016; 16:306. 27329253 10.1186/s12879-016-1625-9 PMC4915108

[51] Meintjes G Lawn SD Scano F; International Network for the Study of HIV-Associated IRIS Tuberculosis-associated immune reconstitution inflammatory syndrome: case definitions for use in resource-limited settings. Lancet Infect Dis 2008; 8:516–23. 18652998 10.1016/S1473-3099(08)70184-1 PMC2804035

