# Supplementary material for: Standardized Methods for Enhanced Quality and Comparability of Tuberculous Meningitis Studies
Source: Clin Infect Dis. 2016 Nov 15;64(4):501–9. doi: 10.1093/cid/ciw757 (PMC5399942; doi:10.1093/cid/ciw757)
Supplement: Supplementary Data [file ciw757_Supplementary_Data.zip › Marais_et_al_Consensus_TBM_Core_Dataset_FINAL_300916.doc]

**Patient demographic data**

| Date of birth  (If day and month not known, please enter year only) | [__\|__]/[__\|__]/[__\|__] *(dd/mm/yy)* |
| --- | --- |
| Patient gender at birth | ⭘ male ⭘female |
| Nationality/ethnicity | [__________________________________] |
| Medical Centre details | PRE-PRINT: Country, City, Hospital |
| Date of admission | [__\|__]/[__\|__]/[__\|__] *(dd/mm/yy)* |
| Hospital registration number on admission | [__\|__\|__\|__\|__\|__] |

Past medical history

**Diabetes**

Does the patient have diabetes? ⭘ Yes ⭘ No ⭘ Not sure

If the answer is ‘yes’:

a. Is the patient using insulin? ⭘ Yes ⭘ No ⭘ Not sure
b. Is the patient using other medication? ⭘ Yes ⭘ No ⭘ Not sure
c. Please describe here what medication:

[______________________________________________________________]

d. What is the most recent HbA1c result? [__|__].[__]%

**HIV status**

What are the results of the HIV test? ⭘ negative ⭘ positive ⭘ not done

What is the date of the HIV test-results: [__|__]/[__|__]/[__|__] *(dd/mm/yy)*

*If the answer to question 7 is ‘positive’ please fill in the following questions*

a. Is the patient on antiretroviral treatment (ART)? ⭘ Yes ⭘ No ⭘ Not sure
b. When did the patient start ART? [__|__]/[__|__]/[__|__] *(dd/mm/yy)* ⭘ Not sure
c. What ART regimen is the patient taking? ⭘ regimen 1 (SITE DEPENDANT)

⭘ regimen 2

⭘ regimen 3

d. What is the date of first positive HIV test [__|__]/[__|__]/[__|__] *(dd/mm/yy)* ⭘ Not sure
e. What is the latest CD4 count? [__|__|__]cells/ml

date: [__|__]/[__|__]/[__|__] *(dd/mm/yy)*

f. What is the lowest (nadir) CD4 count recorded? [__|__|__]cells/ml

date: [__|__]/[__|__]/[__|__] *(dd/mm/yy)*

Is there a history of intravenous drug use? ⭘ Yes ⭘ No ⭘ Not sure

Was the patient BCG vaccinated?

*(or does the patient have a BCG scar)*	⭘ Yes ⭘ No ⭘ Not sure

*If ‘yes’ please answer*

a. Was the vaccination <2 years ago? ⭘ Yes ⭘ No ⭘ Not sure

Was there a recent close TB contact? ⭘ Yes ⭘ No ⭘ Not sure

*If ‘yes’ please answer*

a. Is the source case known to have drug resistant TB? ⭘ Yes ⭘ No ⭘ Not sure

**Previous TB treatment**

Did the patient have TB in the past? ⭘ Yes ⭘ No ⭘ Not sure

If ‘yes’, how many episodes? [__|__]

Did the patient receive TB treatment in the past? ⭘ Yes ⭘ No ⭘ Not sure

*If ‘yes’, please answer the following questions*

a. What was the number of previous TB episodes? [__|__]
b. What is the date of the most recent past episode of TB treatment?

Start date: [__|__]/[__|__]/[__|__] *(dd/mm/yy)* End date: [__|__]/[__|__]/[__|__] *(dd/mm/yy)*

c. What type of TB disease was it?

⭘ smear positive pulmonary TB	⭘smear negative pulmonary TB	⭘ extrapulmonary TB

*If extrapulmonary TB please specify*

🞏 Pleural

🞏 Miliary

🞏 Lymphnode

🞏 Meningeal

🞏 Bone, joint

🞏 Bone, vertebral

🞏 Gastrointestinal TB (including abdominal)

🞏 Genitourinary TB

🞏 Other (specify) [_________________________________]

d. What drugs were used, and what duration?

🞏 Rifampicin	[__|__] months

🞏 Isoniazid	[__|__] months

🞏 Pyrazinamide	[__|__] months

🞏 Ethambutol	[__|__] months

🞏 Streptomycin	[__|__] months

Second line

🞏 Kanamycin	[__|__] months

🞏 Amikacin	[__|__] months

🞏 Capreomycin	[__|__] months

🞏 Levofloxacin	[__|__] months

🞏 Moxifloxacin	[__|__] months

🞏 PAS	[__|__] months

🞏 Cycloserine	[__|__] months

🞏 Ethionamide	[__|__] months

🞏 Protionamide	[__|__] months

🞏 Terizidone	[__|__] months

Is the patient currently on TB treatment? ⭘ Yes ⭘ No ⭘ Not sure

(before TBM diagnosis made/TBM specific treatment started)

*If’ yes’ please specify treatment and duration*

a. Date started: [__|__]/[__|__]/[__|__] *(dd/mm/yy)*
b. Treatment regimen:

🞏 Rifampicin	[__|__] months

🞏 Isoniazid	[__|__] months

🞏 Pyrazinamide	[__|__] months

🞏 Ethambutol	[__|__] months

🞏 Streptomycin	[__|__] months

Second line

🞏 Kanamycin	[__|__] months

🞏 Amikacin	[__|__] months

🞏 Capreomycin	[__|__] months

🞏 Levofloxacin	[__|__] months

🞏 Moxifloxacin	[__|__] months

🞏 PAS	[__|__] months

🞏 Cycloserine	[__|__] months

🞏 Ethionamide	[__|__] months

🞏 Protionamide	[__|__] months

🞏 Terizidone	[__|__] months

**Previous TB preventive therapy (IPT)**

Did the patient receive IPT? ⭘ Yes ⭘ No ⭘ Not sure

*If ‘yes’ please specify*

a. Number of preventive therapy episodes [__|__]
b. Date most recent preventive therapy episode started and ended

[__|__]/[__|__]/[__|__] *(dd/mm/yy)*

**In children <10 years**

Does the patient have BCG vaccination/scar? ⭘ Yes ⭘ No ⭘ Not sure

Was there a recent close TB contact? ⭘ Yes ⭘ No ⭘ Not sure

*(i.e. in the past 2 years)*

*If ‘yes’ please answer the following question*

a. Is the source case known to have drug resistant TB? ⭘ Yes ⭘ No ⭘ Not sure

**Presenting general symptoms and signs**

Temperature (maximum recorded in preceding 24h)	[__|__].[__] °C

Blood pressure (on date of recording, most representative measurement) [__|__|__] / [__|__|__] mmHg

Weight	🞏 measured	OR 🞏estimated	[__|__|__] kg

Height	🞏 measured	OR 🞏estimated	[__|__|__] cm

Weight loss *(In children - Failure to thrive)*	⭘ Yes	⭘ No	⭘ Not sure

Night sweats >2 weeks	⭘ Yes	⭘ No	⭘ Not sure

Cough >2 weeks	⭘ Yes	⭘ No	⭘ Not sure

*Only in children <5 years old;*

Head circumference	[__|__|__] cm

Failure to thrive	⭘ Yes	⭘ No	⭘ Not sure

Is there any clinical evidence of extraneural TB during this episode of disease?

(any other form of TB than TB meningitis)

🞏 yes	🞏 No

*If”yes” please indicate what form (tick one or more if appropriate)*

🞏 Pulmonary

🞏 Pleural

🞏 Miliary

🞏 Lymphnode

🞏 Meningeal

🞏 Bone (⭘joint or ⭘ vertebral)

🞏 Gastrointestinal TB

🞏Genitourinary TB

🞏 Other (specify) [_________________________________]

Is this diagnosis confirmed microbiologically (extraneural sample smear or culture positive?) 🞏 yes	🞏 No

Is there imaging evidence of extraneural TB? 🞏 yes	🞏 No

**Presenting neurological symptoms and signs**

| Date of first neurological symptom  (e.g. headache, focal signs, convulsions) | | [__\|__]/[__\|__]/[__\|__] *(dd/mm/yy)* | |
| --- | --- | --- | --- |
| Fever ⭘ Yes ⭘ No ⭘ Not sure  Headache ⭘ Yes ⭘ No ⭘ Not sure  Vomiting ⭘ Yes ⭘ No ⭘ Not sure  Seizures ⭘ Yes ⭘ No ⭘ Not sure  *If present:*   a. *Generalized* ⭘ Yes ⭘ No b. *Focal* ⭘ Yes ⭘ No | | | Duration  [__\|__\|__] days  [__\|__\|__] days  [__\|__\|__] days  [__\|__\|__] days |
| Cranial nerve palsy (tick any that are present, may be more than 1)  ⭘ Yes ⭘ No ⭘ Not sure | | a. [__] left 3 / 4 / 5 / 6 / 7 b. [__] right 3 / 4 / 5 / 6 / 7 c. [__] other d. [__] none | |
| Hemiplegia/paresis ⭘ Yes ⭘ No ⭘ not known   a. side? ⭘ left ⭘ right b. strength: [__] 0. No contraction 1. Trace of contraction 2. Active movement if eliminated gravity 3. Active movement against gravity 4. Active movement against some resistance 5. Normal power | | | |
| Paraplegia/paresis ⭘ Yes ⭘ No ⭘ not known  Urinary retention ⭘ Yes ⭘ No | | | |
| Tetraplegia/paresis ⭘ Yes ⭘ No ⭘ not known | | | |
| Generalized convulsions ⭘ Yes ⭘ No  Were convulsions witnessed by medical staff? ⭘ Yes ⭘ No | | | |
| Neckstiffness ⭘ yes ⭘ no ⭘ not known | | | |
| Papilledema ⭘ yes ⭘ no ⭘ not known | | | |
| **Glasgow Coma Score (adults and older children)**  Date of recording: [__\|__]/[__\|__]/[__\|__\|__\|__]   a. **Eye opening**   spontaneous 4  to speech 3  to pain 2  none 1   b. **Motor**   Follow commands 6  Localizes pain 5  Withdraws to pain 4  Abnormal flexion 3  Abnormal extension 2  None 1 | | c. **Verbal**   oriented 5  confused 4  inappropriate words 3  moans 2  none 1   d. **score GCS [__\|__] / 15**   ***(a + b + c)***  patient ventilated: 🞏yes 🞏 no  patient sedated: 🞏yes 🞏 no | |
| **Pre-verbal children (infants)** | | | |
| **Modified Glascow Coma Scale***  Date of recording: [__\|__]/[__\|__]/[__\|__\|__\|__]   a. **Eye opening**   Spontaneous 4  To speech 3  To pain 2  None 1   b. **Motor**   Moves spontaneously 6  Withdraws to touch 5  Withdraws to pain 4  Abnormal flexion 3  Abnormal extension 2  None 1 | c. **Verbal**   Coos and babbles 5  Irritable cries 4  Cries to pain 3  Moans to pain 2  None 1   d. **score GCS [__\|__] / 15**   ***(a + b + c)***  patient ventilated: 🞏yes 🞏 no  patient sedated: 🞏yes 🞏 no | | |

TBM grade upon enrollment

TBM grade upon admission? [___]

(1) grade I	= GCS 15, no neurological deficiency
(2) grade II = GCS 11- 14 or GCS 15 and neurological deficiency*
(3) grade III = GCS ≤ 10

**any new onset focal neurological signs in the context of this episode of disease*

| **Baseline investigations: peripheral blood** | | | | |
| --- | --- | --- | --- | --- |
|  | | | | |
| Date sample [__\|__]/[__\|__]/[__\|__]*dd/mm/yy* | | | | |
| Serum hemoglobin [__\|__\|__] g/dl  White cell count [__\|__\|__] 10^9^/l   a. Neutrophils [__\|__] % b. Lymphocytes [__\|__] % c. Monocytes [__\|__] % d. Eosinophils [__\|__] %   Serum sodium [__\|__\|__] mmol/l  Serum glucose (random) [__\|__\|__] mg/dl (paired with CSF)  AST [__\|__\|__] U/l  ALT [__\|__\|__] U/l  HBsAg ⭘ positive ⭘ negative ⭘not done  Anti-HCV ⭘ positive ⭘ negative ⭘not done  *If HIV infected*  CD4 cell count [__\|__\|__]cells/ml ⭘not done  HIV viral load [__\|__\|__\|__\|__\|__]copies/ml | | | | |
| Results IGRA/TST ⭘ positive ⭘ negative ⭘not done | | | | |
| **Baseline investigations: cerebrospinal fluid** | | | | |
| Date sample [__\|__]/[__\|__]/[__\|__]*dd/mm/yy*  Type sample ⭘ lumbar puncture ⭘ ventricular drain  Opening pressure [__\|__] cm H_2_O  Appearance   a. ⭘ clear ⭘ very cloudy/pus-like ⭘ mild cloudy/ opalescent ⭘ unknown b. ⭘ colourless ⭘ white ⭘ yellow ⭘ bloody   Volume CSF [__\|__] ml  White cell count [__\|__\|__\|__] cells/µL   a. Neutrophil percentage [__\|__\|__]% b. Lymphocyte percentage [__\|__\|__]%   Total protein [__\|__\|__]mg/dl  CSF glucose [__\|__\|__] mg/dl   a. Paired blood glucose [__\|__\|__] mg/dl   CSF Lactate [__\|__\|__]mmol/l  ZN smear: ⭘ positive ⭘negative ⭘ not done/not known  India ink stain ⭘ positive ⭘negative ⭘ not done/not known  *And/or*  Cryptococcal antigen test ⭘ positive ⭘negative ⭘ not done/not known  TB NAAT: ⭘ positive ⭘negative ⭘ not done/not known  Specify NAAT: 🞏 XPERT MTB/RIF 🞏 other commercial PCR 🞏 in-house test  *If XPERT MTB/RIF positive (MTB detected):*   a. What was the quantification?   ⭘ very low ⭘ low ⭘ intermediate ⭘ high ⭘ very high   b. RIF resistance detected?   ⭘ no ⭘ yes ⭘ indeterminate ⭘error  Mycobacterial culture date of **results**: [__\|__]/[__\|__]/[__\|__] *dd/mm/yy*  ⭘ Mtb isolated ⭘negative ⭘ not done/not known ⭘ other___________  **Mycobacterial drug susceptibility**  Drug susceptibility: date of **results** [__\|__]/[__\|__]/[__\|__] *dd/yy/mm* | | | | |
| RIF  INH  EMB  SM | 🞏 sensitive  🞏 sensitive  🞏 sensitive  🞏 sensitive | 🞏 resistant  🞏 resistant  🞏 resistant  🞏 resistant | 🞏 indeterminate  🞏 indeterminate  🞏 indeterminate  🞏 indeterminate | 🞏 not done  🞏 not done  🞏 not done  🞏 not done |
| **Baseline investigations: other sample** | | | | |
| Type of sample  ⭘ sputum  ⭘ pleural fluid  ⭘ urine  ⭘ blood  ⭘ other [_________________________________________________________________]  Date sample [__\|__]/[__\|__]/[__\|__]*dd/mm/yy* | | | | |
| ZN smear: ⭘ positive ⭘negative ⭘ not done/not known  TB NAAT: ⭘ positive ⭘negative ⭘ not done/not known  Specify NAAT 🞏 XPERT MTB/RIF 🞏 other commercial PCR 🞏 in-house test  *If XPERT MTB/RIF positive (MTB detected):*   a. What was the quantification?   ⭘ very low ⭘ low ⭘ intermediate ⭘ high ⭘ very high   b. RIF resistance detected?   ⭘ no ⭘yes ⭘ indeterminate ⭘error  Mycobacterial culture date of **results**: [__\|__]/[__\|__]/[__\|__] *dd/mm/yy*  ⭘ Mtb isolated ⭘negative ⭘ not done/not known ⭘ other___________  **Mycobacterial drug susceptibility**  Drug susceptibility: date of **results** [__\|__]/[__\|__]/[__\|__] *dd/yy/mm* | | | | |
| RIF  INH  EMB  SM | 🞏 sensitive  🞏 sensitive  🞏 sensitive  🞏 sensitive | 🞏 resistant  🞏 resistant  🞏 resistant  🞏 resistant | 🞏 indeterminate  🞏 indeterminate  🞏 indeterminate  🞏 indeterminate | 🞏 not done  🞏 not done  🞏 not done  🞏 not done |

| **Patient management** | | | |
| --- | --- | --- | --- |
| **TB Drug**  ADD MULTIPLE RECORDS  IF REQUIRED | **Start/restart**  *(day/month/year)* | **stop**  (day/month/year) | **Reason for stopping** |
| 🞏 Rifampicin dose [__\|__\|__]mg  Rifampicin dose [__\|__\|__]mg | [__\|__]/[__\|__]/[__\|__]  [__\|__]/[__\|__]/[__\|__] | [__\|__]/[__\|__]/[__\|__]  [__\|__]/[__\|__]/[__\|__] | 🞏 (S)AE number [__\|__] 🞏 schedule 🞏 patient initiative 🞏 other  🞏 (S)AE number [__\|__] 🞏 schedule 🞏 patient initiative 🞏 other |
| 🞏 Isoniazid dose [__\|__\|__] mg Isoniazid dose [__\|__\|__] mg | [__\|__]/[__\|__]/[__\|__]  [__\|__]/[__\|__]/[__\|__] | [__\|__]/[__\|__]/[__\|__]  [__\|__]/[__\|__]/[__\|__] | 🞏 (S)AE number [__\|__] 🞏 schedule 🞏 patient initiative 🞏 other  🞏 (S)AE number [__\|__] 🞏 schedule 🞏 patient initiative 🞏 other |
| 🞏 Pyrazinamid [__\|__\|__] mg  Pyrazinamid [__\|__\|__] mg | [__\|__]/[__\|__]/[__\|__]  [__\|__]/[__\|__]/[__\|__] | [__\|__]/[__\|__]/[__\|__]  [__\|__]/[__\|__]/[__\|__] | 🞏 (S)AE number [__\|__] 🞏 schedule 🞏 patient initiative 🞏 other  🞏 (S)AE number [__\|__] 🞏 schedule 🞏 patient initiative 🞏 other |
| 🞏 Streptomycin [__\|__\|__] mg  Streptomycin [__\|__\|__] mg | [__\|__]/[__\|__]/[__\|__]  [__\|__]/[__\|__]/[__\|__] | [__\|__]/[__\|__]/[__\|__]  [__\|__]/[__\|__]/[__\|__] | 🞏 (S)AE number [__\|__] 🞏 schedule 🞏 patient initiative 🞏 other  🞏 (S)AE number [__\|__] 🞏 schedule 🞏 patient initiative 🞏 other |
| 🞏 Ethambutol [__\|__\|__] mg  Ethambutol [__\|__\|__] mg | [__\|__]/[__\|__]/[__\|__]  [__\|__]/[__\|__]/[__\|__] | [__\|__]/[__\|__]/[__\|__]  [__\|__]/[__\|__]/[__\|__] | 🞏 (S)AE number [__\|__] 🞏 schedule 🞏 patient initiative 🞏 other  🞏 (S)AE number [__\|__] 🞏 schedule 🞏 patient initiative 🞏 other |
| 🞏 Levofloxacin [__\|__\|__] mg  🞏 Moxifloxacin [__\|__\|__] mg | [__\|__]/[__\|__]/[__\|__]  [__\|__]/[__\|__]/[__\|__] | [__\|__]/[__\|__]/[__\|__]  [__\|__]/[__\|__]/[__\|__] | 🞏 (S)AE number [__\|__] 🞏 schedule 🞏 patient initiative 🞏 other  🞏 (S)AE number [__\|__] 🞏 schedule 🞏 patient initiative 🞏 other |
| 🞏 Kanamycin [__\|__\|__] mg  🞏 Amikacin [__\|__\|__] mg | [__\|__]/[__\|__]/[__\|__]  [__\|__]/[__\|__]/[__\|__] | [__\|__]/[__\|__]/[__\|__]  [__\|__]/[__\|__]/[__\|__] | 🞏 (S)AE number [__\|__] 🞏 schedule 🞏 patient initiative 🞏 other  🞏 (S)AE number [__\|__] 🞏 schedule 🞏 patient initiative 🞏 other |
| 🞏 Ethionamide [__\|__\|__] mg  🞏 Prothionamide [__\|__\|__] mg | [__\|__]/[__\|__]/[__\|__]  [__\|__]/[__\|__]/[__\|__] | [__\|__]/[__\|__]/[__\|__]  [__\|__]/[__\|__]/[__\|__] | 🞏 (S)AE number [__\|__] 🞏 schedule 🞏 patient initiative 🞏 other  🞏 (S)AE number [__\|__] 🞏 schedule 🞏 patient initiative 🞏 other |
| 🞏 Cycloserine [__\|__\|__] mg | [__\|__]/[__\|__]/[__\|__] | [__\|__]/[__\|__]/[__\|__] | 🞏 (S)AE number [__\|__] 🞏 schedule 🞏 patient initiative 🞏 other |
| 🞏 Capreomycin [__\|__\|__] mg | [__\|__]/[__\|__]/[__\|__] | [__\|__]/[__\|__]/[__\|__] | 🞏 (S)AE number [__\|__] 🞏 schedule 🞏 patient initiative 🞏 other |
| 🞏 Other [__\|__\|__] mg  [__________________________] | [__\|__]/[__\|__]/[__\|__] | [__\|__]/[__\|__]/[__\|__] | 🞏 (S)AE number [__\|__] 🞏 schedule 🞏 patient initiative 🞏 other |

| **Anti inflammatory drugs** | **Start/restart**  *(day/month/year)* | **stop**  (day/month/year) | **Reason for stopping** |
| --- | --- | --- | --- |
| 🞏 Dexamethasone [__\|__\|__] mg  Dexamethasone [__\|__\|__] mg  Dexamethasone [__\|__\|__] mg  Dexamethasone [__\|__\|__] mg | [__\|__]/[__\|__]/[__\|__]  [__\|__]/[__\|__]/[__\|__]  [__\|__]/[__\|__]/[__\|__]  [__\|__]/[__\|__]/[__\|__] | [__\|__]/[__\|__]/[__\|__]  [__\|__]/[__\|__]/[__\|__]  [__\|__]/[__\|__]/[__\|__]  [__\|__]/[__\|__]/[__\|__] | 🞏 (S)AE number [__\|__] 🞏 schedule 🞏 patient initiative 🞏 other  🞏 (S)AE number [__\|__] 🞏 schedule 🞏 patient initiative 🞏 other  🞏 (S)AE number [__\|__] 🞏 schedule 🞏 patient initiative 🞏 other  🞏 (S)AE number [__\|__] 🞏 schedule 🞏 patient initiative 🞏 other |
| 🞏 Aspirin [__\|__\|__\|__] mg  Aspirin [__\|__\|__\|__] mg | [__\|__]/[__\|__]/[__\|__]  [__\|__]/[__\|__]/[__\|__] | [__\|__]/[__\|__]/[__\|__]  [__\|__]/[__\|__]/[__\|__] | 🞏 (S)AE number [__\|__] 🞏 schedule 🞏 patient initiative 🞏 other  🞏 (S)AE number [__\|__] 🞏 schedule 🞏 patient initiative 🞏 other |
| 🞏 Other [__\|__\|__] mg  [____________________] | [__\|__]/[__\|__]/[__\|__] | [__\|__]/[__\|__]/[__\|__] | 🞏 (S)AE number [__\|__] 🞏 schedule 🞏 patient initiative 🞏 other |

| **ART drugs*** | **Start/restart**  *(day/month/year)* | **stop**  (day/month/year) | **Reason for stopping** |
| --- | --- | --- | --- |
| 🞏 REGIMEN 1 | [__\|__]/[__\|__]/[__\|__] | [__\|__]/[__\|__]/[__\|__] | 🞏 (S)AE number [__\|__] 🞏 schedule 🞏 patient initiative 🞏 other |
| 🞏 REGIMEN 2 | [__\|__]/[__\|__]/[__\|__] | [__\|__]/[__\|__]/[__\|__] | 🞏 (S)AE number [__\|__] 🞏 schedule 🞏 patient initiative 🞏 other |
| 🞏 REGIMEN 3 | [__\|__]/[__\|__]/[__\|__] | [__\|__]/[__\|__]/[__\|__] | 🞏 (S)AE number [__\|__] 🞏 schedule 🞏 patient initiative 🞏 other |
| 🞏 INDIVIDUAL AGENTS | [__\|__]/[__\|__]/[__\|__] | [__\|__]/[__\|__]/[__\|__] | 🞏 (S)AE number [__\|__] 🞏 schedule 🞏 patient initiative 🞏 other |
| 🞏 INDIVIDUAL AGENTS | [__\|__]/[__\|__]/[__\|__] | [__\|__]/[__\|__]/[__\|__] | 🞏 (S)AE number [__\|__] 🞏 schedule 🞏 patient initiative 🞏 other |
| 🞏 SECOND LINE | [__\|__]/[__\|__]/[__\|__] | [__\|__]/[__\|__]/[__\|__] | 🞏 (S)AE number [__\|__] 🞏 schedule 🞏 patient initiative 🞏 other |
| 🞏 SECOND LINE | [__\|__]/[__\|__]/[__\|__] | [__\|__]/[__\|__]/[__\|__] | 🞏 (S)AE number [__\|__] 🞏 schedule 🞏 patient initiative 🞏 other |
| **regimens need to be specified in text when results are reported* | | | |

**Hydrocephalus management**

🞏 Medical intervention with diuretics	start date: [__|__]/[__|__]/[__|__] dd/mm/yy	stop date [__|__]/[__|__]/[__|__] dd/mm/yy

Describe [______________________________________________________________________________________________________________]

🞏 Surgical shunting	start date: [__|__]/[__|__]/[__|__] dd/mm/yy

Describe [________________________________________________________________________________________________________________]

| **Patient monitoring** | | | | | | | |
| --- | --- | --- | --- | --- | --- | --- | --- |
| Day of enrollment is day 0 – Week 0 | | | | | | | |
| Day | Day 0 enter study | __/__/___  Day month year | __/__/___  Day/month/year | __/__/___  Day/month/year | __/__/___  Day/month/year | __/__/___  Day/month/year | __/__/___  Day/month/year |
| GCS* /15 | **[__\|__]** | **[__\|__]** | **[__\|__]** | **[__\|__]** | **[__\|__]** | **[__\|__]** | **[__\|__]** |
| **modified GCS should be applied in pre-verbal children* | | | | | | | |
| Ventilated | 🞏 | 🞏 | 🞏 | 🞏 | 🞏 | 🞏 | 🞏 |
| Sedated | 🞏 | 🞏 | 🞏 | 🞏 | 🞏 | 🞏 | 🞏 |
| O_2_ saturation | [__\|__\|__] | [__\|__\|__] | [__\|__\|__] | [__\|__\|__] | [__\|__\|__] | [__\|__\|__] | [__\|__\|__] |
| Blood pressure | [__\|__\|__]/  [__\|__\|__] | [__\|__\|__]/  [__\|__\|__] | [__\|__\|__]/  [__\|__\|__] | [__\|__\|__]/  [__\|__\|__] | [__\|__\|__]/  [__\|__\|__] | [__\|__\|__]/  [__\|__\|__] | [__\|__\|__]/  [__\|__\|__] |

Week 1

| Day | __/__/___  Day/month/year | __/__/___  Day/month/year | __/__/___  Day/month/year | __/__/___  Day/month/year | __/__/___  Day/month/year | __/__/___  Day/month/year | __/__/___  Day/month/year |
| --- | --- | --- | --- | --- | --- | --- | --- |
| GCS /15 | **[__\|__]** | **[__\|__]** | **[__\|__]** | **[__\|__]** | **[__\|__]** | **[__\|__]** | **[__\|__]** |
| Ventilated | 🞏 | 🞏 | 🞏 | 🞏 | 🞏 | 🞏 | 🞏 |
| Sedated | 🞏 | 🞏 | 🞏 | 🞏 | 🞏 | 🞏 | 🞏 |
| O_2_ saturation | [__\|__\|__] | [__\|__\|__] | [__\|__\|__] | [__\|__\|__] | [__\|__\|__] | [__\|__\|__] | [__\|__\|__] |
| Blood pressure | [__\|__\|__]/  [__\|__\|__] | [__\|__\|__]/  [__\|__\|__] | [__\|__\|__]/  [__\|__\|__] | [__\|__\|__]/  [__\|__\|__] | [__\|__\|__]/  [__\|__\|__] | [__\|__\|__]/  [__\|__\|__] | [__\|__\|__]/  [__\|__\|__] |

Week 2

| Day | __/__/___  Day/month/year | __/__/___  Day/month/year | __/__/___  Day/month/year | __/__/___  Day/month/year | __/__/___  Day/month/year | __/__/___  Day/month/year | __/__/___  Day/month/year |
| --- | --- | --- | --- | --- | --- | --- | --- |
| GCS /15 | **[__\|__]** | **[__\|__]** | **[__\|__]** | **[__\|__]** | **[__\|__]** | **[__\|__]** | **[__\|__]** |
| Ventilated | 🞏 | 🞏 | 🞏 | 🞏 | 🞏 | 🞏 | 🞏 |
| Sedated | 🞏 | 🞏 | 🞏 | 🞏 | 🞏 | 🞏 | 🞏 |
| O_2_ saturation | [__\|__\|__] | [__\|__\|__] | [__\|__\|__] | [__\|__\|__] | [__\|__\|__] | [__\|__\|__] | [__\|__\|__] |
| Blood pressure | [__\|__\|__]/  [__\|__\|__] | [__\|__\|__]/  [__\|__\|__] | [__\|__\|__]/  [__\|__\|__] | [__\|__\|__]/  [__\|__\|__] | [__\|__\|__]/  [__\|__\|__] | [__\|__\|__]/  [__\|__\|__] | [__\|__\|__]/  [__\|__\|__] |

Week 3

| Day | __/__/___  Day/month/year | __/__/___  Day/month/year | __/__/___  Day/month/year | __/__/___  Day/month/year | __/__/___  Day/month/year | __/__/___  Day/month/year | __/__/___  Day/month/year |
| --- | --- | --- | --- | --- | --- | --- | --- |
| GCS /15 | **[__\|__]** | **[__\|__]** | **[__\|__]** | **[__\|__]** | **[__\|__]** | **[__\|__]** | **[__\|__]** |
| Ventilated | 🞏 | 🞏 | 🞏 | 🞏 | 🞏 | 🞏 | 🞏 |
| Sedated | 🞏 | 🞏 | 🞏 | 🞏 | 🞏 | 🞏 | 🞏 |
| O_2_ saturation | [__\|__\|__] | [__\|__\|__] | [__\|__\|__] | [__\|__\|__] | [__\|__\|__] | [__\|__\|__] | [__\|__\|__] |
| Blood pressure | [__\|__\|__]/  [__\|__\|__] | [__\|__\|__]/  [__\|__\|__] | [__\|__\|__]/  [__\|__\|__] | [__\|__\|__]/  [__\|__\|__] | [__\|__\|__]/  [__\|__\|__] | [__\|__\|__]/  [__\|__\|__] | [__\|__\|__]/  [__\|__\|__] |

Week 4

| Day | __/__/___  Day/month/year | __/__/___  Day/month/year | __/__/___  Day/month/year | __/__/___  Day/month/year | __/__/___  Day/month/year | __/__/___  Day/month/year | __/__/___  Day/month/year |
| --- | --- | --- | --- | --- | --- | --- | --- |
| GCS /15 | **[__\|__]** | **[__\|__]** | **[__\|__]** | **[__\|__]** | **[__\|__]** | **[__\|__]** | **[__\|__]** |
| Ventilated | 🞏 | 🞏 | 🞏 | 🞏 | 🞏 | 🞏 | 🞏 |
| Sedated | 🞏 | 🞏 | 🞏 | 🞏 | 🞏 | 🞏 | 🞏 |

Week 5

| Day | __/__/___  Day/month/year | __/__/___  Day/month/year | __/__/___  Day/month/year | __/__/___  Day/month/year | __/__/___  Day/month/year | __/__/___  Day/month/year | __/__/___  Day/month/year |
| --- | --- | --- | --- | --- | --- | --- | --- |
| GCS /15 | **[__\|__]** | **[__\|__]** | **[__\|__]** | **[__\|__]** | **[__\|__]** | **[__\|__]** | **[__\|__]** |
| Ventilated | 🞏 | 🞏 | 🞏 | 🞏 | 🞏 | 🞏 | 🞏 |
| Sedated | 🞏 | 🞏 | 🞏 | 🞏 | 🞏 | 🞏 | 🞏 |

Week 6

| Day | __/__/___  Day/month/year | __/__/___  Day/month/year | __/__/___  Day/month/year | __/__/___  Day/month/year | __/__/___  Day/month/year | __/__/___  Day/month/year | __/__/___  Day/month/year |
| --- | --- | --- | --- | --- | --- | --- | --- |
| GCS /15 | **[__\|__]** | **[__\|__]** | **[__\|__]** | **[__\|__]** | **[__\|__]** | **[__\|__]** | **[__\|__]** |
| Ventilated | 🞏 | 🞏 | 🞏 | 🞏 | 🞏 | 🞏 | 🞏 |
| Sedated | 🞏 | 🞏 | 🞏 | 🞏 | 🞏 | 🞏 | 🞏 |

Week 7

| Day | __/__/___  Day/month/year | __/__/___  Day/month/year | | __/__/___  Day/month/year | __/__/___  Day/month/year | __/__/___  Day/month/year | | __/__/___  Day/month/year | __/__/___  Day/month/year |
| --- | --- | --- | --- | --- | --- | --- | --- | --- | --- |
| GCS /15 | **[__\|__]** | **[__\|__]** | | **[__\|__]** | **[__\|__]** | **[__\|__]** | | **[__\|__]** | **[__\|__]** |
| Ventilated | 🞏 | 🞏 | | 🞏 | 🞏 | 🞏 | | 🞏 | 🞏 |
| Sedated | 🞏 | 🞏 | | 🞏 | 🞏 | 🞏 | | 🞏 | 🞏 |
| **a. Eyes** – spontaneous (4), to speach (3), to pain (2), none (1) | | | **b. motor –** follows commands (6), localizes pain (5), withdraws to pain (4), abnormal flexion (3), abnormal extension (2), none (1) | | | | **c. verbal –** oriented (5), confused (4), inappropriate words (3), moans (2), none (1) | | |
| **Score GCS = a + b + c** | | | | | | | | | |
| Follow-up TBM grade?  *(7 and 14 days after initiation TBM treatment)* [___] | | | | | | | | | |
| (1) grade I = GCS 15, no neurological deficiency (2) grade II = GCS 11- 14 or GCS 15 and neurological deficiency (3) grade III = GCS ≤ 10 | | | | | | | | | |

| **Imaging** | | |
| --- | --- | --- |
| Baseline scan 🞏  Follow up scan 🞏 | | |
| Chest X-ray | [__\|__]/[__\|__]/[__\|__]  day month year  not done 🞏 | ⭘ normal  ⭘ miliary pattern  ⭘ other abnormalities suggestive of pulmonary TB |
| MRI-brain | [__\|__]/[__\|__]/[__\|__]  day month year  not done 🞏  contrast enhanced?  🞏 yes 🞏 no | ⭘ normal  ⭘ hydrocephalus  🞏 periventricular oedema  🞏 herniation  ⭘ pre contrast basal hyperintensity  ⭘ basal meningeal enhancement  ⭘ tuberculoma  🞏 single  🞏 multiple  ⭘ infarction  🞏 single  🞏 multiple  Location [____________________________] |
| CT-brain (baseline) | [__\|__]/[__\|__]/[__\|__]  day month year  not done 🞏  contrast enhanced?  🞏 yes 🞏 no | ⭘ normal  ⭘ hydrocephalus  🞏 periventricular oedema  🞏 herniation  ⭘ pre contrast basal hyperintensity  ⭘ basal meningeal enhancement  ⭘ tuberculoma  🞏 single  🞏 multiple  ⭘ infarction  🞏 single  🞏 multiple  Location [____________________________] |
| MRI spine | [__\|__]/[__\|__]/[__\|__]  day month year  not done 🞏  contrast enhanced?  🞏 yes 🞏 no | ⭘ normal  ⭘ abnormalities suggestive of spinal TB  Descriptive findings  [_______________________________________  _______________________________________  ________________________________________  ________________________________________  ________________________________________] |
| Air encephalogram | [__\|__]/[__\|__]/[__\|__]  day month year  not done 🞏 | ⭘ communicating hydrocephalus  ⭘ non-communicating hydrocephalus  ⭘ other [_______________________________] |
|  |  |  |

**Adverse events**

**Adverse event (AE) number** *AE* [__|__]

please fill in a new event form for each event and number events sequentially

Date of ONSET of AE: [__|__]/[__|__]/[__|__] *(dd/mm/yy)*

| **Specify New ADVERSE Event** | |
| --- | --- |
| ⭘ Worsening level of consciousness  *(Fall in GCS ≥2points, for ≥48hrs)* | Likely explanation:  ⭘ raised intracranial pressure  ⭘ infarction  ⭘ tuberculoma  ⭘unknown  ⭘ other [________________________________] |
| ⭘ New focal neurological sign  *(lasting >24 hours)* | Type:  ⭘ hemiplegia  ⭘ paraplegia  ⭘ monoplegia  ⭘ cerebral nerve palsy  ⭘ blindness  ⭘ deafness  ⭘ seizures  ⭘ worsening headache  ⭘ other  [______________________________________] |
| ⭘ Hyponatraemia (<130mmol/l)?  *If yes - lowest value recorded* | [__\|__\|__] mmol/l |
| ⭘ Hypoglycaemia (<2.8mg/dl)?  *If yes - lowest value recorded* | [__\|__\|__] mg/dl |
| ⭘ HIV Stage 4 illness  *(other than TBM)*  **please list as appropriate in setting* | Type:  ⭘ PCP  ⭘ recurrent pneumonia  ⭘ etc* |
| ⭘ Neurological IRIS  *(list criteria used to diagnose)* | Management:  ⭘ ART interruption  ⭘ steroid treatment  ⭘ other [________________________________] |
| ⭘ Other (Specify): [________________________________________________________________] | |

**Grade the event	*(What grade was the event at its worst moment*?*)***

⭘ Grade 1 or 2: asymptomatic, mild, minimal; local or noninvasive intervention indicated

⭘ Grade 3 or 4: requiring or prolonging hospitalization, or life threatening

**Is the event an SAE?**	⭘yes	⭘no	reason[___]

update reason if changed at end of event ( for example if results in death) [___]

(by definition all clinical grade 3 and 4 AEs are considered SAEs)

Reasons why and AE is considered an SAE:

1. Causes death,
2. Life-threatening event (the subject was at immediate risk of death at the time of the event; it does not refer to an event which hypothetically might have caused death if it were more severe)
3. Inpatient hospitalization or prolongation of existing hospitalization
4. Persistent or significant disability/incapacity (a substantial disruption of a person's ability to conduct normal life functions),
5. Congenital anomaly/birth defect
6. Important medical event that may not be immediately life-threatening or result in death or hospitalization but may jeopardize the patient or may require intervention to prevent one of the other outcomes listed in the definition above.

**Is the event an USAE?**	⭘yes	⭘no

**Specify the relatedness to TBM disease:**

🞎 related to TBM

🞎 possibly related to TBM

🞎 not related to TBM

**Specify the relatedness to antituberculous treatment (ATT):**

🞎 related to ATT

🞎 possibly related to ATT

🞎 not related to ATT

**Specify the relatedness to antiretroviral treatment (ART):**

🞎 related to ART

🞎 possibly related to ART

🞎 not related to ART

Date of END of AE: [__|__]/[__|__]/[__|__] *(dd/mm/yy) (not clinically relevant anymore)*

**What was the final outcome of this AE?**

⭘ AE Recovered	⭘ Patient Died	⭘ Condition on-going at end of study

**Outcome**

Assessment Date:	[__|__]/[__|__]/[__|__] *(dd/mm/yy)*

*This form should be filled in at (at least) 1, 6 and 12 months after enrollment*

Study Day:

Month 1	🞏

Month 6	🞏

Month 12	🞏

Other	🞏

Was the patient discharged from hospital? ⭘ Yes	⭘ No

*If ‘yes’ please note date of discharge:* [__|__]/[__|__]/[__|__] *(dd/mm/yy)*

Has the patient died?	⭘ Yes	⭘ No

*If ‘yes’ please note date of death:* [__|__]/[__|__]/[__|__] *(dd/mm/yy)*

*If ‘yes’ what (in the opinion of the attending physician) was the primary cause of death:*

⭘ tuberculous meningitis

⭘ other (please describe) [_______________________________________________________________________]

Was the patient lost to follow up? ⭘ Yes	⭘ No

*If ‘yes’ please note date last known to be alive:* [__|__]/[__|__]/[__|__] *(dd/mm/yy)*

**Glasgow Coma Score** [__|__]

**Disability**

**ADULTS**

**Two simple Questions:**

Does the patient require help from anybody for everyday activities? *(For example eating, drinking, washing, brushing teeth, going to the toilet.)* ⭘ yes	⭘ no

Has the illness left you with any other problems?	⭘ yes	⭘ no

**Modified Rankin Scale**

Please choose which sentence is most appropriate for your current situation: [___]

0. I have no problems
1. I have some small problems, but can have a normal life-style
2. I have some problems that interfere with my life-style, but I can take care of myself
3. I have some problems that interfere with my lifestyle and prevent independent living
4. I have problems that clearly prevent independent living, but do not need constant care.
5. I have problems that make me totally dependant on others and need care day and night.

**Glasgow Outcome Scale**

Choose the condition most appropriate to the condition of the patient at time of assessment [___]

| (1) Death | Severe injury or death without recovery of consciousness |
| --- | --- |
| (2) Persistent vegetative state | Severe damage with prolonged state of unresponsiveness and a lack of higher mental functions |
| (3) Severe disability | Severe injury with permanent need for help with daily living |
| (4) Moderate disability | No need for assistance in everyday life, employment is possible but may require special equipment. |
| (5) Low disability | Light damage with minor neurological and psychological deficits. |

**CHILDREN < 5 years**

Paediatric Glasgow Outcome Score - Extended [__|__]

Review standard questions/algorithm to finalize the score*

8 - Death

7 - Vegetative State (VS)

6 - Lower Severe Disability (Lower SD)

5 - Upper Severe Disability (Upper SD)

4 - Lower Moderate Disability (Lower MD)

3 - Upper Moderate Disability (Upper MD)

2 - Lower Good Recovery (Lower GR)

1 - Upper Good Recovery (Upper GR)

***Algorithm/Questions for Paediatric Glasgow outcome scale-extended**

NOTE: Only problems that have developed or become markedly worse since developing TB meningititis should be considered when completing the GOS-E Peds. That is, the child’s premorbid status must be weighed when answering all questions.

**1. CONSCIOUSNESS**

1a) Is the child able to obey simple commands or say any words?

For younger patients - Can he or she act/react/interact beyond reflexes?

No - Vegetative State (VS) – ***Score 7***

Yes - Skip to 2

*An individual who shows the ability to obey even simple commands or utter any word or communicate specifically in any other way is no longer considered to be in a vegetative state. Eye movements are not reliable evidence of meaningful responsiveness; corroborate with nursing staff and the child’s parents when possible. Confirmation of VS requires full assessment as in the Royal College of Physician Guidelines. However, for infants, actively following the movement of a parent or people/object with eyes, grasping for objects, making faces, etc. are interactions; breast feeding and crying continuously can be reflexes.*

**2. INDEPENDENCE IN THE HOME**

2a) Is the assistance of another person at home essential every day for some activities of daily living?

For younger patients - Is the child dependent upon a caretaker more so than is expected based on age?

Yes - Go to 2b

No - Skip to 3

*For an older child, complete independence and a ‘no’ answer should mean that the person can get washed, put on clean clothes without prompting, prepare food for themselves, deal with callers, and handle minor domestic crises. The person should be able to carry out activities without needing prompting or reminding, and should be capable of being left alone for an age appropriate period. Young children should be able to accomplish age appropriate developmentalmilestones without assistance (see Vineland Daily Living Skills). If a child compensates for physical disability to the point where developmental milestones are only mildly compromised, question parents to determine the level of independence (i.e., A child with a hemiparesis who is still able to complete tasks that other children of that age can be rated as independent).*

2b) Does the child need frequent help or for someone to be around at home most of the time?

For younger patients - Does the child need frequent help from a caretaker to accomplish tasks that a child this age should be able to accomplish (If child sometimes functions at an age appropriate level, then answer ‘no’)?

Yes - Lower Severe Disability – ***Score 6***

No – Upper Severe Disability – ***Score 5***

**3. INDEPENDENCE OUTSIDE THE HOME**

3a) Is the child able to shop and travel without assistance?

For younger patients - Does the child behave age appropriately outside the home?

No - Upper Severe Disability– Score 5

Yes - Skip to 4

*This item considers activities such as shopping and traveling, always in the context of age appropriate behaviors. This includes being able to plan what to buy, take care of money, and behave appropriately in public. The individual need not normally shop, but must be able to do so. A younger child must behave age appropriately in public. An older child may drive or use public transit to get around. Ability to*

*use a taxi is sufficient, provided the person can phone for it themselves and give instruction the driver. Older children who sometimes were allowed to travel independently before the injury should be able to walk to a neighbor’s house, take a school bus, ride a bike, or take public transportation.*

**4. SCHOOL/WORK**

4a) Can the child function at work or in school at his or her previous capacity?

Yes - Skip to 5

No - Go to 4b

*If an adolescent was working before the injury, then his or her current capacity for work should be at the same level. If the individual was seeking work before, then the injury should not have adversely affected chances of obtaining work or the level of work for which he or she is eligible. If the patient was in preschool or a student before the injury, then capacity for school work and school activities should not be adversely affected.*

4b) Level of restriction:

i) Able to work only in a sheltered workshop or non-competitive job, in a school setting for severely impaired children or tutored at home, or currently unable to work or go to school.

Yes - Lower Moderate Disability – ***Score 4***

No - Go to 4b ii

ii) Reduced work or school capacity.

Yes - Upper Moderate Disability – ***Score 3***

**5. SOCIAL & LEISURE ACTIVITIES**

5a) Is the child able to resume regular social and leisure activities?

Yes - Skip to 6

No - Go to 5b

*The individual may not have resumed all previous leisure activities, but should not be prevented from doing so by physical or mental impairment. If he or she has stopped the majority of activities because of loss of interest or motivation, then this is considered a disability. For younger children, social and leisure activities can include games and toys played with caretakers, siblings or other children as well as the ability to interact in a playful manner with others.*

5b) What is the extent of restrictions on social and leisure activities?

i) Unable to participate: Rarely, if ever, take part.

Yes - Lower Moderate Disability – ***Score 4***

No - Go to 5b ii

ii) Participate much less: Less than half as often.

Yes - Upper Moderate Disability – ***Score 3***

No - go to 5b iii

iii) Participate a bit less: At least half as often as before injury.

Yes - Lower Good Recovery – ***Score 2***

No - Skip to 6

**6. FAMILY & FRIENDSHIPS**

6a) Are there psychological problems that have resulted in ongoing disruption with respect to either family or friendships?

Yes - Go to 6b

No - Skip to 7

*Typical personality changes: quick temper, irritability, anxiety, aggressive acts, insensitivity to others, mood swings, depression, and unreasonable or childish behavior that is not age appropriate.*

6b) What is the extent of disruption or strain?

Constant – daily and intolerable; Lower Moderate Disability – ***Score 4***

Frequent – once a week or more, but tolerable; Upper Moderate Disability – ***Score 3***

Occasional – less than weekly; Lower Good Recovery – ***Score 2***

**7. RETURN TO NORMAL LIFE**

7a) Are there any other problems relating to the injury that affect daily life?

Yes - Lower Good Recovery – ***Score 2***

No - Upper Good Recovery – ***Score 1***

**Note: Validated for children with traumatic head injury, further validation required in tuberculous meningitis**

| Laboratory monitoring Assessment Date: [__\|__]/[__\|__]/[__\|__] *(dd/mm/yy)* *This form should be filled for each planned study assessment date* Study Day: Screening 🞏 Day 3 🞏 Week 5 🞏 Month 3 🞏 Day 7 🞏 Week 6 🞏 Month 4 🞏 Week 2 🞏 Week 7 🞏 Month 5 🞏 Week 3 🞏 Week 8 🞏 Month 6 🞏 Week 4 🞏 Unscheduled 🞏 If ‘unscheduled’ what was the reason? [________________________________] **Peripheral blood** | |
| --- | --- |
| Date (dd/mm/yy) | [__\|__]/[__\|__]/[__\|__] |
| Haemoglobin | [__\|__].[__] g/dL Not done🞏 |
| White cells | [__\|__].[__] K/µL or [__\|__].[__] g/L Not done🞏 |
| Neutrophils | [__\|__].[__] % Not done🞏 |
| Lymphocytes | [__\|__].[__] % Not done🞏 |
| Thrombocytes | [__\|__].[__] K/µL or [__\|__].[__] g/L Not done🞏 |
| Date (dd/mm/yy) | [__\|__]/[__\|__]/[__\|__] |
| Na | [__\|__\|__].[__] mmol/L Not done🞏 |
| K | [__\|__].[__] mmol/L Not done🞏 |
| Creatinine | [__\|__\|__].[__] µmol/L Not done🞏 |
| Bilirubin | [__\|__].[__] µmol/L Not done🞏 |
| AST | [__\|__\|__\|__] U/L Not done🞏 |
| ALT | [__\|__\|__\|__] U/L Not done🞏 |
| Glucose | [__\|__].[__] mmol/L Not done🞏 |
| Protein (total) | [__\|__].[__] g/L Not done🞏 |
| Albumin | [__\|__].[__] g/L Not done🞏 |

*HIV infected patients only*

|  | Date (dd/mm/yy) | [__\|__]/[__\|__]/[__\|__] |
| --- | --- | --- |
|  | CD4+ T cell count | [__\|__\|__\|__] cells/ µL Not done🞏 |
| **CSF follow up sample** | | |
|  | Date (dd/mm/yy) [__\|__]/[__\|__]/[__\|__] Not done🞏  Volume CSF [__\|__] ml  White cell count [__\|__\|__\|__] cells/µL   c. Neutrophil percentage [__\|__\|__]% d. Lymphocyte percentage [__\|__\|__]%   Total protein [__\|__\|__](mg/dl)  CSF glucose [__\|__\|__] (mg/dl)   b. Paired blood glucose [__\|__\|__] (mg/dl)   ZN smear: ⭘ positive ⭘negative ⭘ not done/not known  TB NAAT: ⭘ positive ⭘negative ⭘ not done/not known  Date (dd/mm/yy) [__\|__]/[__\|__]/[__\|__] Not done🞏  Specify NAAT 🞏 XPERT MTB/RIF 🞏 other commercial PCR 🞏 in-house test  If XPERT MTB/RIF positive (MTB detected):  What was the quantification? ⭘ very low ⭘ low ⭘ intermediate ⭘ high ⭘ very high  RIF resistance detected? ⭘ no ⭘yes ⭘ indeterminate ⭘error  Mycobacterial culture date of **sample**: (day/month/year) [__\|__]/[__\|__]/[__\|__]  Mycobacterial culture date of **results**: (day/month/year) [__\|__]/[__\|__]/[__\|__]  Culture results: ⭘ Mtb isolated ⭘negative ⭘ not done/not known ⭘ other___________  Drug susceptibility: date of **sample** (day/month/year) [__\|__]/[__\|__]/[__\|__]  Drug susceptibility: date of **results** (day/month/year) [__\|__]/[__\|__]/[__\|__]    DST results:  RIF 🞏 sensitive 🞏 resistant 🞏 indeterminate 🞏 not done  INH 🞏 sensitive 🞏 resistant 🞏 indeterminate 🞏 not done  EMB 🞏 sensitive 🞏 resistant 🞏 indeterminate 🞏 not done  SM 🞏 sensitive 🞏 resistant 🞏 indeterminate 🞏 not done  PZA 🞏 sensitive 🞏 resistant 🞏 indeterminate 🞏 not done | |
